# Analysis of Intervention Strategies for Inhalation Exposure to Polycyclic Aromatic Hydrocarbons and Associated Lung Cancer Risk Based on a Monte Carlo Population Exposure Assessment Model

**DOI:** 10.1371/journal.pone.0085676

**Published:** 2014-01-08

**Authors:** Bin Zhou, Bin Zhao

**Affiliations:** Department of Building Science, School of Architecture, Tsinghua University, Beijing, PR China; Population Health and Preventive Medicine, Malaysia

## Abstract

It is difficult to evaluate and compare interventions for reducing exposure to air pollutants, including polycyclic aromatic hydrocarbons (PAHs), a widely found air pollutant in both indoor and outdoor air. This study presents the first application of the Monte Carlo population exposure assessment model to quantify the effects of different intervention strategies on inhalation exposure to PAHs and the associated lung cancer risk. The method was applied to the population in Beijing, China, in the year 2006. Several intervention strategies were designed and studied, including atmospheric cleaning, smoking prohibition indoors, use of clean fuel for cooking, enhancing ventilation while cooking and use of indoor cleaners. Their performances were quantified by population attributable fraction (PAF) and potential impact fraction (PIF) of lung cancer risk, and the changes in indoor PAH concentrations and annual inhalation doses were also calculated and compared. The results showed that atmospheric cleaning and use of indoor cleaners were the two most effective interventions. The sensitivity analysis showed that several input parameters had major influence on the modeled PAH inhalation exposure and the rankings of different interventions. The ranking was reasonably robust for the remaining majority of parameters. The method itself can be extended to other pollutants and in different places. It enables the quantitative comparison of different intervention strategies and would benefit intervention design and relevant policy making.

## Introduction

Polycyclic aromatic hydrocarbons (PAHs) are a widespread class of semi-volatile organic compounds (SVOCs) from incomplete combustion of organic matter. They are air pollutants typically found indoors and outdoors [1], with significant sources at both places, including automobile exhaust, fire plant waste, indoor fuel use for cooking or heating, and smoking, etc. Several carcinogenic PAHs have long been known to produce cancer in animals, and epidemiologic studies have shown associations of airborne PAHs with lung cancer among workers in occupational settings [1]–[3]. USEPA has listed sixteen PAHs as priority pollutants to guide the make of control policy and design of research, including: naphthalene (Nap), acenaphthylene (Acy), acenaphthene (Ace), fluorine (Fluo), phenanthrene (Phe), anthracene (Ant), fluoranthene (Flu), pyrene (Pyr), chrysene (Chry), benzo[*a*]anthracene (BaA), benzo[*b*]fluoranthene (BbF), benzo[*k*]fluoranthene (BkF), benzo[*a*]pyrene (BaP), dibenzo[*a*,*h*]anthracene (DBA), indeno[1,2,3-*c*,*d*]pyrene (IP) and benzo[*g*,*h*,*i*]perylene (BghiP) [4].

To reduce the adverse health effect of PAH exposure, various intervention measures have been adopted. But these interventions for inhalation exposure to PAHs, as well as other air pollutants, cannot be easily evaluated; and the evaluation process can be very time-consuming and expensive. Some intervention studies for PAHs were improved stove programs aiming to reduce fuel-related PAH exposure in rural populations [5]–[6] and some others were similar programs conducted in occupational settings [7]. Most of these studies measured PAH metabolites [5]–[7] while a few others used modeling techniques [8]. Modeling methods are an alternative to the measurement-based methods. Although they are less valid in terms of exposure assessment compared with actual measurements, they have their unique strength especially when large scale intervention campaigns cannot be easily done or they are not even feasible due to reasons like unaffordable expenses: this situation is more likely to happen in poorer countries where, however, people are more vulnerable to combustion-related exposures which typically include PAHs.

Stochastic exposure models have been developed to assess population exposure to air pollutants [9]–[12]. Most of the methods adopted a “microenvironment” concept to model population exposure to certain pollutants: individual’s daily activities were categorized according to the places of occurrence (microenvironments, including various indoor environments, commute and outdoor), and with knowledge of the concentrations at each place, total exposure is estimated by the time-weighted sums of exposures at each microenvironment. Either measured concentrations [13] or modeled ones [14] can be used for different microenvironments. If modeled concentrations are used, the method has the potential to provide a quick and affordable way to evaluate various intervention strategies, which can be accomplished by changing one or several of the model parameters to reflect a certain intervention.

With a proven model for PAH population inhalation exposure established in our previous study [14], it is now time to explore the possibility of conducting intervention experiments through models and simulations. This would provide an important method for evaluating indoor air pollution interventions, being a useful complement and ideally even alternative to the commonly used field measurements. In this paper, we are to demonstrate several interventions that can be simulated with this method, and compare the effectiveness of these intervention strategies in terms of PAH inhalation exposure and associated lung cancer risk. These interventions are implemented among the 2006 baseline population of Beijing, China, based on which the model has been practiced and validated [14]. All 16 priority PAHs recommended by USEPA are included in the study.

## Materials and Methods

### Study Population and Baseline Scenarios

A total of 15.81 million residents lived in our study area in the year 2006, consisting of 84% urban and 16% rural residents; other major characteristics of this population were: 51.1% male, 31.1% smokers, 72% employed and 9% school-age children [15]. More detailed information can be found in our previous paper [14].

The overall Beijing population was divided into “Urban” and “Rural” sub-populations, because of the difference in major risk factors (*e*.*g*. use of solid fuels indoors). The “Baseline” scenarios were then defined for these two sub-populations respectively, and were denoted as “Baseline-Urban” (B-u) and “Baseline-Rural” (B-r) scenarios. All parameters in the Baseline scenarios were set to reflect the reality in 2006 in the Beijing region [14].

### Monte Carlo Population Exposure and Associated Lung Cancer Risk Assessment

This study followed the protocol for Monte Carlo (MC) population exposure simulation described in our previous paper [14]. Inhalation exposure to PAHs in a specific microenvironment was the product of concentration, time spent and pulmonary ventilation rate while there. Distributions of indoor PAH concentrations and population exposures were estimated by MC simulation. 10,000 replications were run in each simulation. Oracle Crystal Ball was used for MC simulation.

Population attributable fraction (PAF) was used to quantify the lung cancer risk attributable to PAH exposure. PAF is the proportional reduction in disease or death that would occur if exposure to the risk factor were reduced to a theoretical minimum [16], or zero as is the case here. And it can be calculated by:
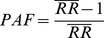
(1)where 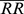 is the average of the calculated relative risks for all simulated individuals in each MC simulation. RR is the relative risk of certain lifetime PAH exposure compared to non-exposed situation, and it was calculated based on the lifetime exposure to equivalent benzo[*a*]pyrene (B[*a*]Peq) concentrations, which are the standardized concentrations using toxicity equivalency factors (TEFs) [17]. The calculation of RR was based on Equation (2), and it was fully described in our previous methodology study [14].
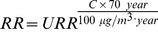
(2)where URR is unit relative risk and it is the relative risk following a lifetime exposure to BaP of 100 µg/m3⋅year, and C is the lifetime e B[a]Peq xposure concentration.

While PAF was used to estimate the risk attributable to PAH exposure in a given scenario, another parameter, potential impact fraction (PIF), was introduced to evaluate the effectiveness of a certain intervention strategy. It is the proportional reduction if associated risk factor were reduced to a non-minimal counterfactual exposure [16], and it can be calculated using the following equation
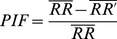
(3)where 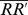 is the average relative risk in the MC simulation of the intervention case.

### Intervention Cases

We analyzed and compared the effectiveness of the intervention strategies listed in Table 1. These cases were designed to address the risks faced by either the urban or the rural sub-populations. The effect of the intervention was quantified comparing with the baseline scenario for each sub-population (urban or rural). The rationales for the interventions and the methods to simulate them are given below.

**Table 1:** Description of intervention cases in the study.

Case Name	Sub-Population	Short Name	Description
Smoking Free	Urban & Rural	SF-u/SF-r	All smoking activity is removed
Clean Fuel	Rural	CF-Hf-r	HALF rural use of coal and biomass is shifted to LPG
		CF-All-r	ALL rural use of coal and biomass is shifted to LPG
Atmospheric Cleaning	Urban	Atm-Hf-u	Outdoor PAH concentration is halved
		Atm-WHO-u	Outdoor PAH concentration is lowered to WHO standard
	Rural	Atm-WHO-r	Outdoor PAH concentration is lowered to WHO standard
Exhaust when Using Indoor Fuel	Urban	Ex-u	Exhaust hoods are used in all urban households
	Rural	Ex-80-r	Only 80% emissions remain indoors
		Ex-50-r	Only 50% emissions remain indoors
		Ex-20-r	Only 20% emissions remain indoors
Indoor PM Cleaner	Urban & Rural	IC-u/IC-r	An average air cleaner for PM is added indoors (no direct effect on gas phase)

About 50% of Chinese men smoked according to a national survey done in 2002 with higher smoking rate in the rural area [18], and the overall smoking rate was 31.1%. According to the same study, less than 20% of urban households and 10% of rural households prohibited smoking completely at their homes, and about 30% of urban work place had such a prohibition. In this intervention scenario, smoking activity was completely removed from all residences and work places. It was simulated by removing smoking-related emissions from the indoor particle and PAH concentration models. Two cases for both urban (Smoking Free-urban, or SF-u) and rural (SF-rural, or SF-r) sub-populations were involved.

Rural households have been shown to suffer from serious pollution from indoor use of solid fuel (coal or biomass) for cooking purposes. The quantity and prevalence of solid fuel use were estimated from a survey conducted in Beijing suburb [19], which were presented in detail in the supplementary material of [14]. In this intervention scenario, cases of two different degrees were studied: either half (Clean Fuel-Half-rural, or CF-Hf-r) or all (CF-All-r) of rural households with solid fuel use were shifted to liquid petroleum gas (LPG), which had much less emissions of particles and PAHs. This intervention was simulated by changing solid fuel use in these households to LPG in the indoor concentration model, which includes the amount of fuel used and its emission of particles and PAHs.

Atmospheric PAH pollution was a very important source of PAH exposure in China. In the model, outdoor PAH concentrations were directly used for both estimating indoor PAH concentration and calculating total inhalation exposure, and the data were collected from measurement studies from 2005–2007 in Beijing in the literature [14]. We provide a brief summary of the medians and standard deviations (SD) of the seasonal averaged concentrations for the sixteen PAH congeners in Table 2. In this scenario, outdoor concentration was first halved from current situation (Atmospheric pollution-Half-urban, or Atm-Hf-u) and then further reduced to WHO guideline level (Atm-WHO-u and Atm-WHO-r). The WHO guideline [19] was designed for BaP only (as an indicator for PAH mixtures), and the highest guideline value (*i.e.* the lowest standard) was 1.2 ng/m^3^ for BaP, which would produce an excess lifetime lung cancer risk of 1/10,000 after lifetime exposure to PAH mixtures containing this amount of BaP (other higher standards, *e.g.* 0.12 or 0.012 ng/m^3^, were deemed too strict for use in China). We estimated the proportion of BaP in the total B[*a*]Peq concentrations in all the atmospheric measurements involved, and used the average value of 0.381 to estimate the corresponding B[*a*]Peq concentration under this guideline.

**Table 2:** Median and standard deviation of the seasonal averaged atmospheric concentrations of PAHs used in this study.

PAHs (ng/m^3^)		Nap	Acy	Ace	Fluo	Phe	Ant	Flu	Pyr
Spring	median	1674.7	1076.3	78.9	122.3	157.5	11.3	79.6	23.2
	SD	3632.8	2447.8	101.6	239.6	393.9	29.0	173.4	38.0
Summer	median	3961.6	695.0	22.0	69.8	96.1	6.6	46.8	9.3
	SD	12071.8	1384.5	21.4	119.5	219.1	17.7	103.8	15.5
Autumn	median	3532.0	780.3	75.6	126.7	104.9	12.8	59.7	14.5
	SD	8626.9	2215.0	93.1	253.0	262.0	37.5	133.6	22.9
Winter	median	1699.3	1638.1	141.4	177.1	331.0	43.0	219.6	62.2
	SD	4071.3	3725.2	149.8	339.7	840.6	115.3	552.6	108.4
**PAHs (ng/m^3^)**		**Chry**	**BaA**	**BbF**	**BkF**	**BaP**	**DBA**	**IP**	**BghiP**
Spring	median	8.8	8.6	6.9	5.5	7.6	1.8	4.5	4.7
	SD	14.5	11.4	14.8	26.1	14.9	3.3	7.7	9.0
Summer	median	7.2	1.3	3.2	1.3	1.3	1.3	2.4	1.8
	SD	12.8	2.1	7.7	6.8	2.1	2.6	4.3	3.7
Autumn	median	8.1	2.8	7.2	2.1	3.1	1.8	4.7	6.1
	SD	14.8	4.0	17.7	9.3	5.0	3.7	8.1	11.1
Winter	median	24.0	19.1	24.3	10.5	13.4	3.9	9.8	13.9
	SD	44.3	27.7	55.2	48.6	21.9	7.3	18.6	22.6

*Note*: SD stands for standard deviation.

Enhancing ventilation during cooking event is a common practice to reduce indoor pollution from cooking fuel use. People either use an exhaust hood (fume extractor) or simply open windows and/or doors for this purpose. In this intervention scenario, we simulated this practice by enlarging the air exchange rates for all urban households who mainly used exhaust hoods (Exhaust-urban, or Ex-u), and also by multiplying an equivalent emission remaining factor (ERF) to current emission rates of cooking activity for rural households who might adopt mixed strategies (using exhaust hoods, installing chimneys and opening window). ERFs of 80% (Exhaust-80-rural, or Ex-80-r), 50% (Ex-50-r) and 20% (Ex-20-r) were used in the intervention cases. In the baseline scenarios, 70% of the urban households only used exhaust hoods “sometimes” or never and 50% emissions were assumed to remain indoors for those who used, while all rural households with coal use (about 11% of the population) used chimneys and 10% emissions were assumed to remain indoors for them [14]. In other situations, all indoor emissions were assumed to remain indoors in the baseline scenarios.

Many of the PAH congeners can attach to airborne particles. Indoor particle cleaner was more and more commonly found in Chinese households to remove indoor particles, and hence particle-bound PAHs. We searched the largest air cleaner provider in China (making up around 80% of the market) and estimated the clean air delivery rate (CADR) of an average particle cleaner used in China. The estimate was the average of all cleaner models weighted by their market shares, which was estimated from the sale records of two popular Chinese online shopping websites in the recent months. Please refer to Table S1 for details of the shares of different particle cleaner models). The estimated average CADR was 134 m^3^/h. In this intervention scenario, all urban (Indoor Cleaner-urban, or IC-u) and rural (IC-r) households were equipped with an average indoor particle cleaner, and it was operated 16 hours a day which was about the typical time people spent at home.

## Results

### Performance of Different Interventions in Terms of Lung Cancer Risks

**Figure 1:**
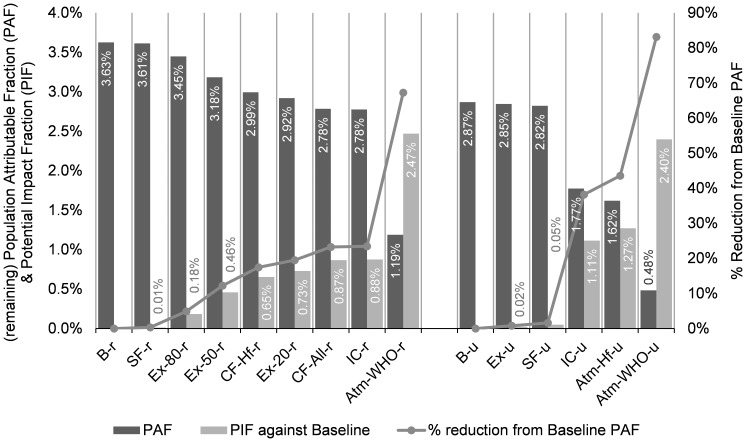
The population attributable fractions (PAFs) (dark column) in the Baseline scenarios, the remaining PAFs (dark column) and their percentage reductions from the Baseline (line), and the potential impact fractions (PIFs) (light column) for different interventions, sorted by PIF values. **Abbreviations:** B-r/B-u: Baseline, rural/urban; SF-r/SF-u: Smoking free, rural/urban; Ex-20-r/Ex-50-r/Ex-80-r: Exhaust while cooking, with 20%/50%/80% remaining indoors, rural; Ex-u: All households use exhaust hoods while cooking, urban; CF-Hf-r/CF-All-r: Clean fuel, Half/All households shift from solid fuel to gas for cooking, rural; IC-r/IC-u: Indoor cleaners are used to removed indoor particles, rural/urban; Atm-WHO-r/Atm-WHO-u: Atmospheric PAH concentrations are reduced to WHO guideline levels, rural/urban; Atm-Hf-u: Atmospheric PAH concentrations are halved from current levels, urban.

Figure 1 illustrates the PAFs of lung cancer risk of inhalation PAH exposures in the baseline scenarios, the remaining PAFs and their percentage reductions from the baseline scenarios in the intervention cases, and the corresponding PIFs. The baseline PAFs are 3.63% for rural households and 2.87% for urban households. The remaining PAFs in the intervention cases range from 3.61% to 1.19% for rural households and 2.85% to 0.48% for urban households, and the corresponding PIFs range from 0.01% to 2.47% for rural households and from 0.02% to 2.40% for urban households.

Atmospheric cleaning (Atm) shows the greatest reductions in PAFs and also the largest PIFs, demonstrating the greatest potential for alleviating associated lung cancer risk. Indoor particle cleaner (IC) is the second most effective intervention in terms of lung cancer risk for both rural and urban households. Smoking prohibition (SF) and adoption of exhaust hoods in urban population (Ex-u) show very small impact on PAH-related lung cancer risk. Clean fuel use (CF) brings appreciable reduction while rather large exhaust rates (Ex) are required to have similar magnitude of impact (Ex-80/50/20-r). Generally, intervention strategies aiming at indoor sources (SF, Ex and CF) perform better among rural households, because of heavier pollution from solid fuel use in rural population. And there is the following rankings in terms of associated lung cancer risk reduction performance: Atm>IC>CF (r)>Ex (r)>SF>Ex (u).

### Comparing the Effectiveness of Different Interventions

**Figure 2:**
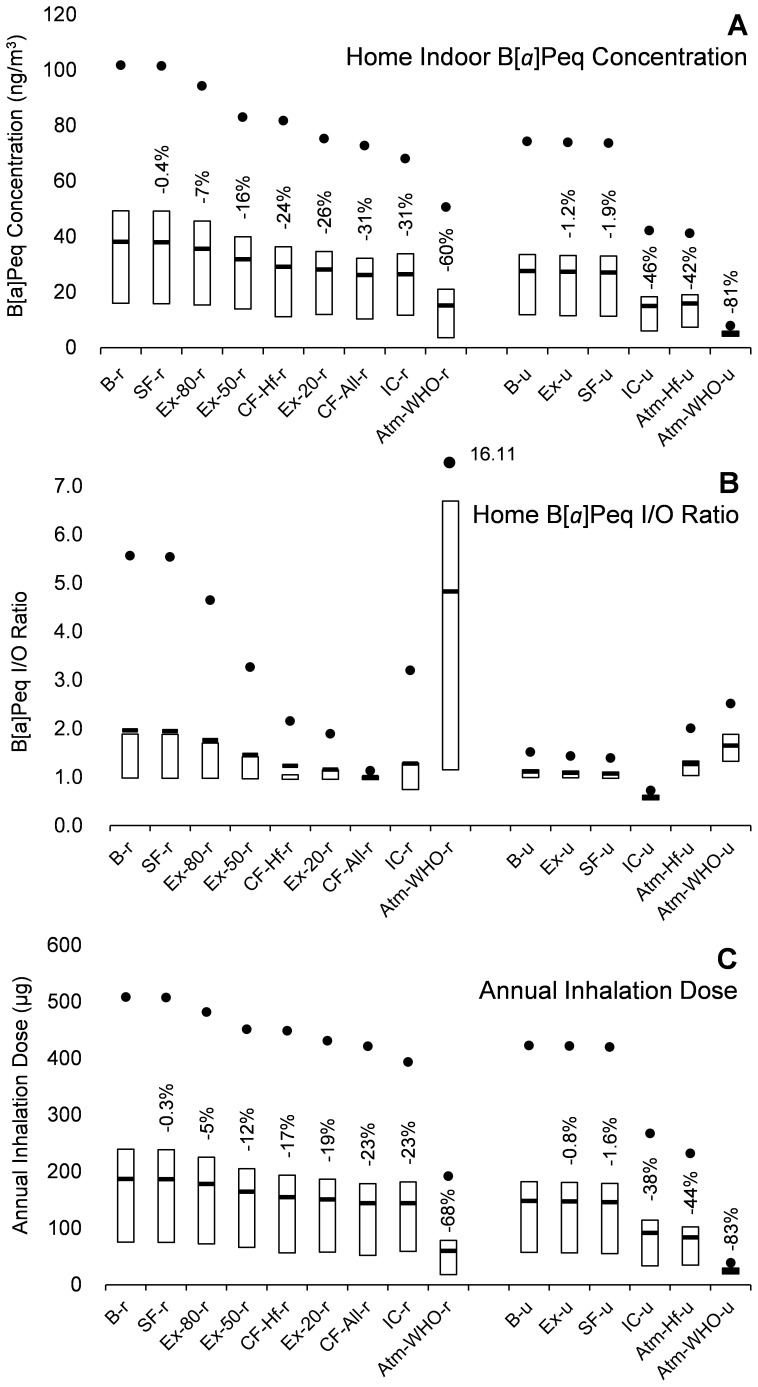
Distributions of three key parameters in different intervention cases and their percentage changes from Baseline scenarios (rural and urban), showing the Means (bar), Interquartile ranges (box) and 95% upper limits (dot) of (A) Home indoor B[*a*]Peq concentrations, sorted by PIF values; (B) Indoor/Outdoor ratios (I/O ratios) for home indoor B[*a*]Peq concentrations; and (C) Annual inhalation dose of B[*a*]Peq. Labels show the percentage reduction of the mean value of a parameter in each intervention case from the corresponding Baseline scenario. **Abbreviations:** B-r/B-u: Baseline, rural/urban; SF-r/SF-u: Smoking free, rural/urban; Ex-20-r/Ex-50-r/Ex-80-r: Exhaust while cooking, with 20%/50%/80% remaining indoors, rural; Ex-u: All households use exhaust hoods while cooking, urban; CF-Hf-r/CF-All-r: Clean fuel, Half/All households shift from solid fuel to gas for cooking, rural; IC-r/IC-u: Indoor cleaners are used to removed indoor particles, rural/urban; Atm-WHO-r/Atm-WHO-u: Atmospheric PAH concentrations are reduced to WHO guideline levels, rural/urban; Atm-Hf-u: Atmospheric PAH concentrations are halved from current levels, urban.

Figure 2 shows the reductions or changes from the baseline scenarios in the Means, Interquartile ranges and 95% upper limits of home indoor B[*a*]Peq concentrations (Panel A), home B[*a*]Peq I/O (indoor concentration over outdoor) ratios (Panel B) and annual inhalation doses (Panel C) for different intervention cases.

Smoking prohibition (SF) again shows very limited impact on all three parameters, while reducing atmospheric PAH concentration (Atm) have the greatest potential for reducing indoor concentration and annual dose (over 60% reduction for rural households and over 80% for urban households). But the “Atm” interventions cannot deal with the pollution from indoor sources, as is indicated by the higher I/O ratios than the baseline scenarios, especially for rural households (Atm-WHO-r). Enhancing ventilation during cooking activities has very small impact for the urban households (Ex-u) but the reduction can be as large as 26% in indoor B[*a*]Peq concentration and 19% in annual inhalation dose for the rural households (Ex-20-r). Adopting an average indoor particle cleaner for rural households (IC-r) has a similar effect in magnitude with substituting all rural use of solid fuel to LPG (CF-All-r), and in these two cases, indoor B[*a*]Peq concentration and annual dose for rural households are very close to those for urban households (B-u) indicating that indoor sources are creating the major gap between rural and urban households (B-r and B-u). Particle cleaner also has a great impact on indoor B[*a*]Peq concentration for urban population (IC-u) which is even greater than the intervention removing 50% of the current outdoor PAH pollution (Atm-Hf-u), but this situation is turned over when comparing the annual dose because IC-u households suffer higher atmospheric pollution when they are outdoor.

The 95% upper limits show similar trends of change with the means, and large 95% upper limits in I/O ratio would mean that significant indoor sources still exist in at least some of the studied households. The only 95% upper limit under 1.0 is found in IC-u, meaning that more than 95% of the population have indoor B[*a*]Peq concentrations lower than in the atmosphere, which is a joint result of small indoor source and use of indoor cleaning device. In these households (with I/O ratio lower than 1.0), people can choose to spend more time indoors to further reduce their annual inhalation dose to PAHs.

### Changes of Exposure Pattern after Intervention

Airborne PAHs can come from either indoor or outdoor sources (denoted with upper case letters: IN or OUT), and exposure to PAHs can also happen either indoors or outdoors (denoted with lower case letters: in or out). Therefore there are four combinations of the source of PAHs and the place of exposure to them: IN-in, IN-out, OUT-in and OUT-out, which stand for “indoor exposure to PAHs of indoor origin”, “indoor exposure to PAHs of outdoor origin”, and so forth. In this study, we do not consider IN-out exposure because emissions from one individual household have limited influence on its own exposure outdoors, compared with emissions from direct outdoor sources and all other households. This concept of exposure pattern was presented and well discussed in [14]. Analysis of the changes of IN-in, OUT-in and OUT-out exposures can help better understand what component of exposure each intervention affects, how much potential for improvement each intervention is aiming at, and how much potential is left after a certain intervention. This analysis can evaluate the design of an intervention and guide further intervention afterwards.

**Figure 3:**
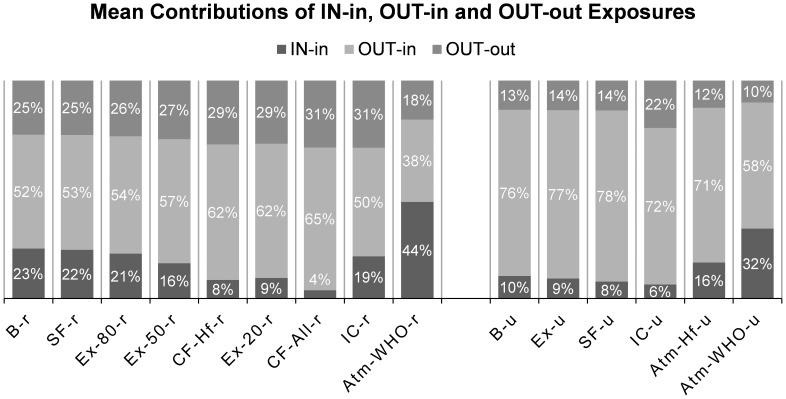
Mean contributions of IN-in, OUT-in and OUT-out exposures for the Baseline scenarios and different intervention cases. **Abbreviations:** B-r/B-u: Baseline, rural/urban; SF-r/SF-u: Smoking free, rural/urban; Ex-20-r/Ex-50-r/Ex-80-r: Exhaust while cooking, with 20%/50%/80% remaining indoors, rural; Ex-u: All households use exhaust hoods while cooking, urban; CF-Hf-r/CF-All-r: Clean fuel, Half/All households shift from solid fuel to gas for cooking, rural; IC-r/IC-u: Indoor cleaners are used to removed indoor particles, rural/urban; Atm-WHO-r/Atm-WHO-u: Atmospheric PAH concentrations are reduced to WHO guideline levels, rural/urban; Atm-Hf-u: Atmospheric PAH concentrations are halved from current levels, urban.

**Figure 4:**
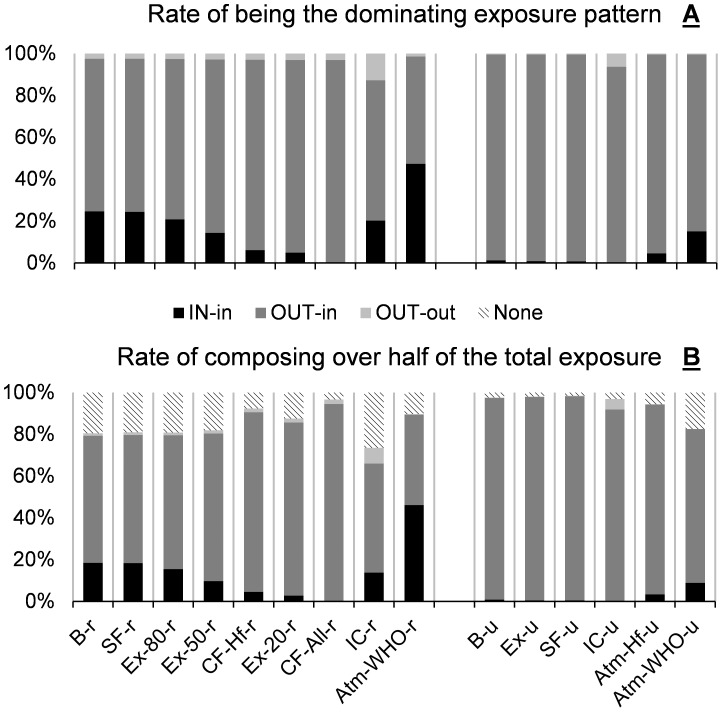
Characteristics of the patterns of the three exposures in different intervention cases and the Baseline scenarios: (A) Rates of IN-in, OUT-in and OUT-out exposures being the dominating exposure pattern; and (B) Rates of IN-in, OUT-in and OUT-out exposures composing over half of the total exposure. **Abbreviations:** B-r/B-u: Baseline, rural/urban; SF-r/SF-u: Smoking free, rural/urban; Ex-20-r/Ex-50-r/Ex-80-r: Exhaust while cooking, with 20%/50%/80% remaining indoors, rural; Ex-u: All households use exhaust hoods while cooking, urban; CF-Hf-r/CF-All-r: Clean fuel, Half/All households shift from solid fuel to gas for cooking, rural; IC-r/IC-u: Indoor cleaners are used to removed indoor particles, rural/urban; Atm-WHO-r/Atm-WHO-u: Atmospheric PAH concentrations are reduced to WHO guideline levels, rural/urban; Atm-Hf-u: Atmospheric PAH concentrations are halved from current levels, urban.

As shown in Figure 3, in average, OUT-in exposure is by far the dominating exposure pattern in all cases expect Atm-WHO-r, in which most outdoor pollution has been eliminated. This observation justifies the findings that outdoor pollution related interventions show greater impact on both indoor B[*a*]Peq concentrations and related lung cancer risks. It can be observed in Figure 3 that, only reducing indoor originated pollution has limited effect of intervention, even for rural households with relatively high prevalence of solid fuel use. However, Figure 4 shows that these interventions (Ex-80-r, Ex-50-r, CF-Hf-r, Ex-20-r, CF-All-r) significantly reduces the number of households with IN-in exposure being the dominating exposure pattern, leaving outdoor originated PAHs being the leading risk factor by far for these families. Further intervention focused indoors would not have any noticeable effect for them, so it is necessary to combine interventions aiming at outdoor originated PAHs with these indoor-focused interventions. IC-r and IC-u are good examples of reducing both indoor and outdoor originated exposures at the same time.

## Discussion

### Comparison with Existing Studies

No studies on the quantitative performance of PAH pollution intervention were found at the time of this study. Existing literature is explored to make a qualitative comparison with some of our simulation results.

Allen et al. [20] reported a 60% reduction in indoor fine particle concentrations following usage of air filters indoors in 25 homes in Canada. Batterman et al. [21] showed that PM levels in child’s bedrooms with a free-standing HEPA filter can be reduced by an average of 50% in 69 homes in the US. Our model demonstrated an average of 46% reduction in indoor B[*a*]Peq concentration for urban residents and 31% for rural residents (Figure 2A). Since the data were for PM rather than PAH and the population was not Chinese, this comparison is only qualitative; but we can still see that the magnitudes of the effect brought by indoor cleaner use are similar.

According to an improved stove program conducted in China’s Xuanwei County [22], improved stove could lead to 20–60% reduction in indoor PM_10_ concentrations or 20–80% in indoor BaP concentrations. Adding chimneys to the stoves would further reduce the concentrations by 40–70% for PM_10_ or 50–90% for BaP. Another group of researchers reported 61% reduction in indoor SO_2_ concentration for intervened households in Guizhou, China, and 38% reduction in CO concentrations [23]. Yet one more study suggested that PM_10_ concentrations were reduced by 88–98% after stove improvement in villages from three Chinese provinces [24]. Our results showed that enhancing ventilation during cooking with solid fuels in rural areas (Ex-r, a scenario similar to the improved stove programs) could reduce indoor B[*a*]Peq concentration by 26% in average (Figure 2A). The higher outdoor concentration in Beijing than in the villages involved above could be one of the reasons that led to the difference between the reported reduction and ours.

A stove intervention study in Peru [5] suggested a median of 47–74% reduction in indoor PM_2.5_ concentrations, while the median reduction in 10 urinary hydroxylate PAH metabolites (OH-PAHs) was reported to be 19%–52%. Two programs of 30 and 27 participating homes respectively were included in the study. The reduction in indoor PM concentration was similar in magnitude to those observed in the aforementioned Chinese studies. Our model predicted a reduction of 12–23% in annual inhalation dose of B[*a*]Peq for different interventions that focused on indoor fuel use in rural areas (Figure 2C). Many factors could lead to its difference from the values reported in literature, and this is only a qualitative comparison limited by the current status of field study on PAH exposure interventions.

### Sensitivity and Uncertainty

**Figure 5:**
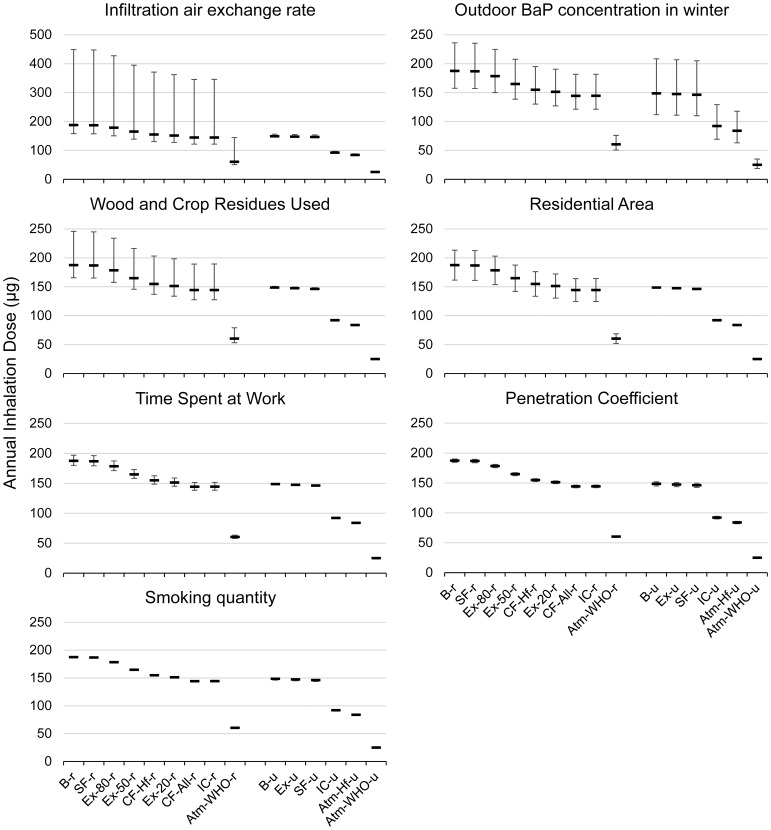
Means (bar) and changes in means corresponding to a +/− standard deviation change in a certain parameter. **Abbreviations:** B-r/B-u: Baseline, rural/urban; SF-r/SF-u: Smoking free, rural/urban; Ex-20-r/Ex-50-r/Ex-80-r: Exhaust while cooking, with 20%/50%/80% remaining indoors, rural; Ex-u: All households use exhaust hoods while cooking, urban; CF-Hf-r/CF-All-r: Clean fuel, Half/All households shift from solid fuel to gas for cooking, rural; IC-r/IC-u: Indoor cleaners are used to removed indoor particles, rural/urban; Atm-WHO-r/Atm-WHO-u: Atmospheric PAH concentrations are reduced to WHO guideline levels, rural/urban; Atm-Hf-u: Atmospheric PAH concentrations are halved from current levels, urban.

The five most sensitive parameters for either urban or rural sub-population as identified in [14], were included in sensitivity analysis; this would thus include seven parameters in total in this analysis. These parameters, their baseline values, standard deviations and rankings for both urban and rural sub-populations are listed in Table 3. The seven parameters were changed in a ±SD range, and the corresponding upper/lower limits in annual inhalation dose to B[*a*]Peq were calculated. The ranges are also listed in Table 3, and the influence on the rankings of intervention strategies is illustrated in Figure 5.

**Table 3:** Ranges of percentage change in annual inhalation dose with a standard deviation change in selected parameters with their baseline values and standard deviations: top five most sensitive parameters for either urban or rural sub-population.

Variable	Baseline (SD), urban^c^	Baseline (SD), rural^c^	Percentage change, urban (%)d,e	Percentage change, rural (%)d,e
Infiltration air exchange rate (1/hour)	0.31 (0.23)	0.59 (0.47)	−0.710/5.19 (2)	−15.6/139.7 (1)
Outdoor BaP concentration in winter (ng/m^3^)^a^	13.4 (21.9)	13.4 (21.9)	40.4/−24.7 (1)	26.0/−15.9 (2)
Residential area (at home) (m^2^)	53.4 (24.6)	128.9 (59.3)	−0.531/1.44 (5)	−11.6/31.2 (3)
Quantity of wood and crop residues used (kg/day)^b^	0	4.00 (2.61)	Not applicable	13.8/−13.8 (4)
Time spent at work/school (hour)	5.45 (3.01)	5.45 (3.01)	−0.035/0.044 (>5)	−3.97/5.09 (5)
Penetration coefficient (at home)	0.90 (0.06)	0.90 (0.06)	2.35/−2.35 (3)	1.52/−1.52 (>5)
Smoking quantity of all family members (#/day)	9 (19)	9 (19)	1.63/−1.63 (4)	0.233/−0.233 (>5)

*^a^*Outdoor concentration of some other PAH congeners could also bring percentage change of over 1% but were omitted for clarity, which included BaP, Nap, DBA, BbF, BkF, Acy in all seasons, and IP, Ant, BaA, Phe, Flu in winter. They all had smaller percentage influence than BaP in winter.

*^b^*Quantity of coal used could also bring percentage change of over 1% but was omitted for clarity. It had smaller percentage influence than the quantity of wood and crop residues used.

*^c^*SD stands for standard deviation, which is listed in brackets.

*^d^*Values separated by “/” are percentage changes when subtracting or adding standard deviations to the baseline values.

*^e^*Rankings of variables for urban and rural sub-populations respectively are listed in brackets; the table is sorted by the larger of the percentage changes in annual inhalation dose for urban and rural sub-populations.

The top five parameters have great influence on the final rankings of the intervention strategies for rural households, while only outdoor PAH concentration (represented by BaP; notes of Table 3 include a full list of the contributing PAH congeners) has significant influence on this ranking for urban households. The sixth and seventh factors (penetration coefficient and smoking quantity of all family members) are already shown to have negligible effect on the general ranking, and thus smaller factors are not included in this analysis. This effect can be observed by the overlapping of the ranges corresponding to a ±SD change in a specific parameter for the cases that are adjacent in ranking. The overlapping of these ranges would suggest a change in ranking if taking the uncertainty in parameters into account, while non-overlapping ranges would suggest a reasonably robust ranking when the input changes in a reasonable range. In other words, apart from the top five parameters included here, the ranking of different intervention strategies is rather robust to different parameter values. Better robustness is observed for intervention rankings among urban households. These results are also a demonstration of the uncertainty associated with the model inputs, stating that the five inputs (or groups of inputs) above are the main source of uncertainty in the simulated ranking of different interventions. Further discussions on the model uncertainty can be found in the section “Limitations”.

### Changes in the Modeled Lung Cancer Risk with Different Unit Relative Risk Values

URR value was used in the calculation of relative risk (RR) in Equation (2). Its value has been shown to have large influence on the modeled lung cancer risk as expressed by PAF values [14]. We used the value of 4.49 from a study on Chinese population [25]. The changes in the modeled PAF and PIF values using different URR values are briefly explored here to address the importance of URR in determining lung cancer risk estimate. URR is first changed by ±20% to reveal the sensitivity around the current value, and an alternative URR value of 1.30 for Asian population from a review study [2] is used to illustrate the range of its influence.

It is shown in Table 4 that, the modeled PAFs and PIFs change moderately when URR changes around its current value (4.49). But the risk estimates can change dramatically when the alternative URR (1.30) is used. However, in any of the cases, the ranking of the interventions in terms of either PAF or PIF does not change. Nonetheless, this fact draws the attention to the important role that URR plays in determining risk estimate. The value 4.49 that we used was from a study on a rural population in Xuanwei, China, who were exposed to coal smoke from cooking [26] and the value of 4.49 has been adopted to analyze the lung cancer risk due to inhalation exposure to PAHs in Chinese population [25]. It is therefore considered suitable for the application in the population in this study. However, it can never be too cautious when choosing a value for URR.

**Table 4:** Changes in Modeled PAF and PIF values with different URRs.

Intervention Cases	Modeled PAF				Modeled PIF			
	URR = 4.49	URR +20%	URR −20%	URR = 1.30	URR = 4.49	URR +20%	URR −20%	URR = 1.30
B-r	3.63%	4.07%	3.09%	0.64%	–	–	–	–
SF-r	3.61%	4.05%	3.08%	0.63%	0.01%	0.01%	0.01%	0.00%
Ex-80-r	3.45%	3.87%	2.94%	0.60%	0.18%	0.21%	0.16%	0.03%
Ex-50-r	3.18%	3.57%	2.71%	0.56%	0.46%	0.51%	0.39%	0.08%
CF-Hf-r	2.99%	3.36%	2.55%	0.52%	0.65%	0.73%	0.55%	0.11%
Ex-20-r	2.92%	3.27%	2.49%	0.51%	0.73%	0.82%	0.62%	0.13%
CF-All-r	2.78%	3.12%	2.37%	0.49%	0.87%	0.97%	0.74%	0.15%
IC-r	2.78%	3.11%	2.36%	0.49%	0.88%	0.98%	0.74%	0.15%
Atm-WHO-r	1.19%	1.33%	1.01%	0.21%	2.47%	2.77%	2.10%	0.43%
B-u	2.87%	3.22%	2.44%	0.50%	–	–	–	–
Ex-u	2.85%	3.19%	2.42%	0.50%	0.02%	0.03%	0.02%	0.00%
SF-u	2.82%	3.17%	2.40%	0.49%	0.05%	0.05%	0.04%	0.01%
IC-u	1.77%	1.99%	1.51%	0.31%	1.11%	1.25%	0.95%	0.19%
Atm-Hf-u	1.62%	1.82%	1.38%	0.28%	1.27%	1.43%	1.08%	0.22%
Atm-WHO-r	0.48%	0.54%	0.41%	0.08%	2.40%	2.69%	2.04%	0.42%

*Abbreviations*: B-r/B-u: Baseline, rural/urban; SF-r/SF-u: Smoking free, rural/urban; Ex-20-r/Ex-50-r/Ex-80-r: Exhaust while cooking, with 20%/50%/80% remaining indoors, rural; Ex-u: All households use exhaust hoods while cooking, urban; CF-Hf-r/CF-All-r: Clean fuel, Half/All households shift from solid fuel to gas for cooking, rural; IC-r/IC-u: Indoor cleaners are used to removed indoor particles, rural/urban; Atm-WHO-r/Atm-WHO-u: Atmospheric PAH concentrations are reduced to WHO guideline levels, rural/urban; Atm-Hf-u: Atmospheric PAH concentrations are halved from current levels, urban.

### Implication

This method can quantify the effectiveness of different interventions, and is thus very useful in rational policy making when deciding what intervention to support in reality. And by using parameters suitable for different regions and different pollutants, this model can be extended to evaluate the intervention elsewhere and for other pollutants, as long as appropriate pollutant- and population-specific data are collected, which may include pollutants’ presence in air, emission, demographical and behavioral characteristics of the population, etc.

The results here support that the most effective way to reduce PAH exposure and related risk is to clean the atmosphere. But atmospheric cleaning requires great and integrated effort of the whole society, including reducing industrial and fire plant emissions, reducing automobile emissions and transforming the structure of energy use. Therefore, it is highly infeasible in reality despite of its effectiveness. This gap between the great benefit and the equally great difficulty of enforcement calls for designs of interventions in presence of the current atmospheric pollution level. Indoor cleaner is only one of these choices with demonstrated effects in our study, and further research would benefit the intervention design.

### Limitations

A major limitation comes with the model adopted for indoor PAH concentration estimation. This model has several important assumptions that need to be addressed. First, it is assumed that indoor air is well mixed; thus, one concentration can represent an entire residence. This assumption is justified by the fact that people generally move around in their homes/offices. If the proportion of time spent at different rooms within the house is of interest, models accounting for spatial heterogeneity should be used instead. Further, it is assumed that airborne PAHs follow the linear instantaneous equilibrium assumption, and that indoor emissions are evenly distributed over a 24 hour period. These two assumptions do not significantly bias the calculation of total airborne concentrations, and are therefore suitable for assessing inhalation exposure [14]. However, the proposed model in this study cannot be used to calculate phase-specific PAH concentrations and thus cannot be used to estimate dermal or ingestion exposure [27], and it is why we only studied the inhalation exposure. And since calculated indoor concentrations are averaged values throughout the season, it is only valid for chronic disease; acute disease risk, like acute lower respiratory infections, cannot be assessed using this model framework.

There might be joint changes in other input parameters in the model when simulating a certain intervention by changing one or several input parameters. One typical example is the change in tin/tout to adjust for changes in indoor and outdoor PAH concentrations. For example, in households with I/O ratio far over 1.0 (*e.g.* Atm-WHO-r), people can choose to spend more time outdoors to avoid the heavy indoor pollution. Similarly, those in households with I/O ratio lower than 1.0 may choose to spend more time indoors. This behavioral phenomenon is hard to predict by model but should be acknowledged when using the modeling technique to predict changes in population exposure and risk.

It should also be noted that, the result that smoking prohibition brings little impact on indoor PAH concentration and related exposure and lung cancer risk is restricted to PAHs. It is well-known that cigarette smoke contains a large family of toxics, including nicotine. Comprehensive evaluation of cigarette control effects should involve all these harmful pollutants, although it is beyond the scope of our current study.

In this study, we used 1-stage MC simulation rather than a 2-stage simulation. 1-stage MC simulation has only one loop in calculation, and 2-stage MC simulation extends the 1-stage simulation by adding an extra loop into the existing one. 2-stage simulation can therefore distinguish variability from uncertainty in the distributions of model inputs, and allow for comprehensive uncertainty analysis. We did not incorporate 2-stage uncertainty analysis into this study for clarity considerations: as a pilot study using modeling method for intervention assessment, the addition of uncertainty makes the presentation of the results extremely difficult. In order to keep the paper readable but still convey the core idea, we decided not to involve 2-stage uncertainty in this study. Instead, we conducted the sensitivity analysis based on the 1-stage MC simulation. This sensitivity analysis incorporated the ranges of the model input values and thus could reveal the uncertainty associated with the model and its inputs to some extent. In this manner, the results are scientifically complete and also clear to read. But, of course, a 2-stage MC simulation will provide a more comprehensive understanding of the effectiveness of different interventions, and give more insights of the robustness of the rankings of these interventions.

## Conclusions

In this study, we adopted a Monte Carlo population exposure assessment model to quantify and compare different intervention strategies for inhalation exposure to PAHs and associated lung cancer risk for Beijing population in the year 2006. Our results gave the first application of MC method for intervention analyses, and showed that MC method can be well utilized in this situation. The quantitative results showed that, atmospheric cleaning has the greatest potentials to remove PAH inhalation exposure and related lung cancer risk, while indoor particle cleaner and clean fuel also have appreciable impact on the exposure and risk. The exposure pattern analysis provided rationale behind the relative performance of these interventions, and showed how each intervention affected the different components of the total inhalation exposure. The rankings of these strategies are relatively robust with most of the input parameters expect the infiltration air exchange rate, outdoor PAH concentration, residential area, the amount of solid fuels used indoors and the time spent at different microenvironments. This robustness should still be further checked with a more comprehensive 2-stage MC simulation.

## Supporting Information

Table S1 **Market shares of different indoor particle cleaners of the top Chinese air cleaner provider (YADU), estimated from the online sale records in two major Chinese online shopping websites (TAOBAO.com and 360BUY.com).**
(DOC)
