# Supplementary material for: Analysis of Intervention Strategies for Inhalation Exposure to Polycyclic Aromatic Hydrocarbons and Associated Lung Cancer Risk Based on a Monte Carlo Population Exposure Assessment Model
Source: PLoS One. 2014 Jan 8;9(1):e85676. doi: 10.1371/journal.pone.0085676 (PMC3885750; doi:10.1371/journal.pone.0085676)
Supplement: Table S1 — Market shares of different indoor particle cleaners of the top Chinese air cleaner provider (YADU), estimated from the online sale records in two major Chinese online shopping websites (TAOBAO.com and 360BUY.com). (DOC) [file pone.0085676.s001.doc]

***Supporting Information***

**Analysis of Intervention Strategies for Inhalation Exposure to Polycyclic Aromatic Hydrocarbons and Associated Lung Cancer Risk Based on a Monte Carlo Population Exposure Assessment Model**

Bin Zhou, Bin Zhao*

Department of Building Science, School of Architecture, Tsinghua University, Beijing 100084, PR China

**Table S1. Market shares of different indoor particle cleaners** of the top Chinese air cleaner provider (YADU), estimated from the online sale records in two major Chinese online shopping websites (TAOBAO.com and 360BUY.com)

| **Model** | **CADR** | **Monthly Sale & Shares** | | **Weighted Average Shares** |
| --- | --- | --- | --- | --- |
|  |  | **TAOBAO.com** | **360BUY.com** |  |
| **Total** | **134 m^3^/h** | **998** | **388** |  |
| KJF2202T | 115 m^3^/h | 23.8% | 6.9% | 19.1% |
| KJG1201S | 92 m^3^/h | 16.8% | 10.1% | 15.0% |
| KJF2901 | 199 m^3^/h | 12.6% | 13.6% | 12.9% |
| KJF2903E | 199 m^3^/h | 10.7% | 8.2% | 10.0% |
| KJF2203E | 115 m^3^/h | 2.3% | 22.0% | 7.8% |
| KJG1202 | 92 m^3^/h | 3.5% | 12.2% | 5.9% |
| KJF4902 | 307 m^3^/h | 6.1% | 0.0% | 4.4% |
| KJG2104 | 115 m^3^/h | 0.0% | 12.8% | 3.6% |
| KJG1282 | 92 m^3^/h | 4.0% | 0.0% | 2.9% |
| KJF2801N | 100 m^3^/h | 3.7% | 0.0% | 2.7% |
| KJG881 | 23 m^3^/h | 2.3% | 1.7% | 2.1% |
| KJG200W | 115 m^3^/h | 2.3% | 0.4% | 1.8% |
| KJG240C | 115 m^3^/h | 0.0% | 6.1% | 1.7% |
| KJG200AS | 115 m^3^/h | 2.0% | 0.0% | 1.4% |
| KJG130AS | 92 m^3^/h | 1.6% | 0.7% | 1.3% |
| KJF2203 | 115 m^3^/h | 1.6% | 0.0% | 1.2% |
| KJG2702 | 199 m^3^/h | 0.5% | 2.1% | 1.0% |
| KJG230S | 115 m^3^/h | 0.1% | 2.7% | 0.8% |
| KJF883 | 46 m^3^/h | 1.0% | 0.0% | 0.7% |
| KJG300AS | 199 m^3^/h | 0.8% | 0.0% | 0.6% |
| KJF2801S | 100 m^3^/h | 0.7% | 0.0% | 0.5% |
| KJG2701 | 199 m^3^/h | 0.5% | 0.0% | 0.4% |
| KJG3001AS | 199 m^3^/h | 0.5% | 0.0% | 0.4% |
| KJF2902 | 199 m^3^/h | 0.5% | 0.0% | 0.4% |
| KJF1282 | 92 m^3^/h | 0.5% | 0.0% | 0.4% |
| KJG2101 | 115 m^3^/h | 0.4% | 0.0% | 0.3% |
| KJG882 | 31 m^3^/h | 0.2% | 0.6% | 0.3% |
| KJG882C | 31 m^3^/h | 0.2% | 0.0% | 0.1% |
| KJF883C | 31 m^3^/h | 0.2% | 0.0% | 0.1% |
| KJG2102 | 115 m^3^/h | 0.2% | 0.0% | 0.1% |
| KJG1801N | 100 m^3^/h | 0.2% | 0.0% | 0.1% |
| *Note*: Depending on the availability of the sales data in the two websites, different methods were used to obtain the monthly sales for them. For TAOBAO.com, monthly sales were obtained for September to October, and for 360BUY.com, monthly sales were obtained by monthly averages of the total sales for each model.  CADR: clean air delivery rate | | | | |
